# Frequent short sickness absence, occupational health service utilisation and long-term sickness absence due to mental disorders among young employees

**DOI:** 10.1007/s00420-021-01728-5

**Published:** 2021-06-06

**Authors:** Jaakko Harkko, Hilla Nordquist, Olli Pietiläinen, Kustaa Piha, Minna Mänty, Tea Lallukka, Ossi Rahkonen, Anne Kouvonen

**Affiliations:** 1 Faculty of Social Sciences, University of Helsinki, Helsinki, Finland; 2 Department of Public Health, Faculty of Medicine, University of Helsinki, Helsinki, Finland; 3 South Eastern Finland, University of Applied Sciences, Kotka, Finland; 4 Unit of Strategy and Research, City of Vantaa, Vantaa, Finland; 5 Research Institute of Psychology, SWPS University of Social Sciences and Humanities, Wroclaw, Poland; 6 Administrative Data Research Centre, Northern Ireland, Centre for Public Health, Queen’s University, Belfast, UK

**Keywords:** Work disability, Occupational health primary care, Frequent attenders, Cox regression, Municipal employees, Socioeconomic inequality

## Abstract

**Objectives:**

We examined whether frequent short-term sickness absence (FSTSA) and primary care use in occupational health service (OHS) were associated with medically-certified long-term sickness absence (LTSA) due to mental disorders among young employees.

**Methods:**

We used record-linkage data covering the young employees (< 35 years) of the City of Helsinki, Finland (*n* = 8,282) from 2010 to 2014. The outcome was LTSA due to mental disorders. Cox regression models were fitted.

**Results:**

FSTSAs were associated with subsequent LTSA. Also OHS use predicted LTSA due to mental disorders; however, this association was not found for those with prior FSTSA.

**Conclusions:**

Both FSTSA and primary care use indicate subsequent LTSA independently, and together these indicators identify a larger proportion of individuals at risk of LTSA due to mental disorders.

## Introduction

Mental disorders are increasingly contributing to sickness absence (SA) from work in the economically developed countries (Lidwall et al. 2018; Nicholson 2018; Mauramo et al. 2018; Stansfeld et al. 2011; Bultmann et al. 2005). In Finland, mental disorders are the leading cause of medically-certified long-term sickness absence (LTSA) and disability retirement alongside with musculoskeletal disorders (Kela 2017). Overall LTSA causes large financial burden to employees, employers and societies due to loss of productivity, health care costs, and financial compensation for absence (Nicholson 2018, van Hoffen et al. 2019). At the individual level mental disorders affect the quality of life (Saarni et al. 2007). In addition, LTSA may cause social stigma and isolate the individual from the normal day-to-day life (Nicholson 2018; Baumann 2007). This effect might be more substantial among younger employees (Knapstad et al. 2014). Therefore, the identification and treatment of mental disorders at early stages is very important.

The association between prior SA and future SA has been well-established in previous studies. The length and frequency of prior SA are associated with the risk for subsequent SA (Notenbomer et al 2019; Koopmans et al 2008; Laaksonen et al. 2013). Even short-term SA spells predict future longer SA spells (Laaksonen et al. 2013), also among younger employees and SA due to mental disorders (Sumanen et al. 2017). In addition, previous SA has been found to be a risk factor in studies aiming to develop effective screening tools for all-cause (Airaksinen et al. 2018; Schouten et al. 2016) and mental disorder-related LTSA (van Hoffen et al. 2019). Thus, SA records might be used to identify workers vulnerable to future LTSA. It is also known that frequent attendance (FA) at primary healthcare units is associated with mental disorders (Gili et al. 2011), and that especially persistent FA may indicate underlying chronic psychiatric conditions (Smits et al. 2014). However, it is yet to be determined, whether short-term SA spells and frequent primary care utilisation identify the same or different individuals at risk of LTSA due to mental disorders. From the perspective of targeting preventive measures, the question is whether the identification of at-risk individuals could be increased using linked data obtainable from different electronic health records, such as SA records and medical records.

In Finland, occupational health service (OHS) is a major provider of primary care for working-age population. OHS typically provides primary care services alongside with preventive measures. OHS primary care services are accessible to 90% of the whole of working population, three out of four workers name OHS as their main and preferred type of primary care provider, and the patients of OHS physicians only seldom visit public health centre GPs (Ikonen et al. 2013; Virtanen and Mattila 2011). Hence OHS is the most suitable location to investigate the use of primary care services of the working population in Finland. Recent studies from Finland have found that frequent occupational health service (OHS) utilisation may be associated with higher levels of subsequent SA (Reho et al. 2018; Reho et al. 2020; Harkko et al. 2020). Our study extends the previous research on the associations between frequent short-term sickness absence (FSTSA) spells for any cause and LTSA due to mental disorders by supplementing these analyses with data on primary care utilisation (i.e. OHS utilisation). To our best knowledge, no prior studies have assessed whether and to which extent these indicators are complementary from the perspective of identification of persons at risk of LTSA due to mental disorders.

Several studies from different European countries show that LTSA due to mental disorders is more common among women than men (Lidwall et al. 2018; van Hoffen et al. 2019; Halonen et al. 2018). A Swedish register-based study (Lidwall et al. 2018) with the entire workforce aged 16–64 (*n* = 6 192,397) showed that the risk of SA due to mental disorders exceeding 14 days is highest among those aged 30–39. A Norwegian study with survey and register data (Foss et al. 2010) among 30–45-year-olds (*n* = 8333) with outcome of > 8 weeks SA due to mental disorders showed that the age-related differences exist only among men. Other demographic risk factors were lower socioeconomic position (Foss et al. 2010), and working in health care, education and social services (Lidwall et al. 2018). Moreover, it has been shown that, in Finland, a high use of OHS mostly adheres to these sociodemographic and socioeconomic patterns (Harkko et al. 2020). For these reasons, in this study, we include gender and occupational class in the analyses.

The aim of the present study was to assess whether data on primary care utilisation may enhance the identification of the risk of LTSA due to mental disorders, compared with examining the relationship between FSTSA and subsequent occurrence of LTSA due to mental disorders only. To do so, we examined (1) whether FSTSA associates with subsequent LTSA due to mental disorders, (2) whether OHS visits before and after FSTSA associate with LTSA due to mental disorders, and (3) whether gender, occupational class and disease burden at baseline period affect the examined associations.

## Materials and methods

### Study population and design

Our study focuses on the younger employees of the City of Helsinki, Finland, who all share the same OHS provider, Occupational Health Helsinki. OHS services are free of charge for the employees and access to OHS is equal and typically good, making OHS the main source and the preferred type of primary care for the employees of the City of Helsinki. During the study period, the employees were required to provide a medical certificate for their SA when the length of the absence was more than 3 days. Therefore, it can be assumed that most of the employees with SA have been in contact with the OHS. In addition, OHS monitors the amount of SA of the employees as a part of prevention of occupational health hazards. Moreover, the OHS of the City of Helsinki has consistent policies on treatment of mental disorders. Previous evidence presented above show the relationship between shorter and longer SA, and highlights the importance of preventing the worsening of mental disorders particularly among younger employees.

This is a retrospective cohort study based on administrative records. The study is part of the Helsinki Health Study (HHS), an ongoing cohort study that covers the employees of the City of Helsinki (Lahelma et al. 2013). Helsinki is the capital of Finland and the City of Helsinki is Finland’s largest employer with c. 38,000 employees annually. The City of Helsinki provides a range of public services to its residents, ranging from health care, education and social welfare to public transport, infrastructure and technical services.

The present study population consisted of 18–34-year-old employees with at least 12 months of sustained employment during the years 2010–2014 and with no prior LTSA due to mental disorders before the beginning of the follow-up (*n* = 8,282). We had a full record of LTSA from 2004 onwards resulting individual washout-periods with no prior LTSA to vary between 7 and 12 years. The data determining eligibility were retrieved from the City’s personnel register, and Social Insurance Institution of Finland’s (SII) SA register. The records were linked using national ID numbers assigned to all permanent residents of Finland (Gissler and Haukka 2004).

### Outcome

The outcome was a LTSA (≥ 11 calendar days) due to mental disorders. The outcome was followed-up from the register of SII up to 24 months from the beginning of the follow-up. The data included diagnostic information related to LTSA, and we used SA spells with F-diagnoses in the ICD-10 coding scheme. The beginning of the follow-up period was defined individually in relation to individual’s short-term SA spells within a one-year screening period. The follow-up started from the first day after the end of individual’s index short-term SA spell. The index SA spell was the 6th SA spell occurring within the one-year screening period for those who were defined as FSTSA. For the rest of the participants the index SA spell was the last short-term SA spell within the screening period, or the follow-up began at the end of the screening period when the person had no short-term SA spells. The data were used in single-record/single-failure form. Time-to-event was measured on a daily scale from the start of the follow-up until the event occurrence or the end of the follow-up. The data were retrieved from SII’s sickness allowance register that includes all sickness allowance spells extending over 11 calendar days granted to permanent residents in Finland.

### Predictors

FSTSA was defined as six short-term SA spells (< 11 calendar days) occurring over a one-year time-period due to any reason. Our threshold represents the closest to the upper quartile of the population. It is to be noted that our definition deviates from the more frequently used threshold found in the research literature (three-to-four short-term SA spells/year) (Notenbomer et al. 2019; Koopmans et al 2008; Sumanen et al. 2017). We expect the high number of short-term SA spells to reflect the employer’s policy to allow self-referred SA up until three working days. The data were derived from the City’s personnel register.

OHS primary care visits at baseline were measured as the number of visits one year before the beginning of the follow-up (0, 1–2, 3 or more visits). OHS primary care visits after the short-term SA were measured as the number of visits over the first-4 months during the follow-up. To avoid reverse causation, we included only those OHS visits that occurred before the event (LTSA due to mental disorders). The variable was categorised into three groups: 0 visits, 1–2 visits, and 3 visits or more. The OHS measure reflected all-cause service utilisation and was not limited to service use for mental health reasons. The data related to OHS utilisation were derived from the OHS electronic medical records.

### Covariates

Occupational class as an indicator of socioeconomic position was controlled to take into account the social and economic factors that potentially confound the relationships between FSTSA, OHS utilisation and LTSA due to mental disorders. Occupational class, obtained from the City of Helsinki personnel register, was measured with four categories: “managers and professionals” (e.g. teachers and physicians), “semi-professionals” (e.g. nurses and foremen), “routine non-manuals” (e.g. child care and elderly care workers) and “manual workers” (e.g. tram drivers and care assistants). The data were collected annually thus reflecting the status of the year of the beginning of the follow-up.

Disease burden at baseline period was measured by two variables. We measured the cumulative length of both short-term SA and LTSA spells occurring during the one-year baseline period before the beginning of the follow-up. LTSA spells were classified as 0–11 days, 12–41 days, 42 or more days. The corresponding categories for short-term SA spells were 0 days, 1–13 days and 14 or more days. The data for LTSA were retrieved from SII’s records and for short-term SA from the employer’s records.

### Statistical analysis

Hazard ratios (HRs) with their 95% confidence intervals (95% CIs) for LTSA due to mental disorders were calculated using Cox regression. Kaplan–Meier product limit estimates with survival graphs were produced to present gender differences in the association between FSTSA and LTSA due to mental disorders. We found no interaction effect between gender and FSTSA or OHS utilisation variables, and therefore, the analyses were run using pooled data.

Statistical analyses were carried out to examine the predictive quality of FSTSA and OHS use on the LTSA due to mental disorders, and to investigate how occupational class and disease burden at baseline period affect these relationships. To do so, we sequentially introduced the covariates in regression models. First, the predictors were introduced adjusted for age and gender (Model 1). Second, Model 2 includes baseline OHS and FSTSA variables together, i.e. model 2 represents the proportion of the effect that is independent of the other potential indicators. At the following stages, occupational class (Model 3) and disease burden at baseline (Model 4) were added to model 2. We applied two distinct modelling strategies to produce the estimates for OHS use before and after SAs. First, in the analyses of the effect of FSTSA and baseline OHS use on LTSA, the estimates were produced for all participants. In preliminary analyses, we found a statistically significant interaction effect between FSTSA and OHS follow-up utilisation variables. For the analytical purposes, due to their temporal order, we assumed non-FSTSA and FSTSA as distinct subpopulations. As only one subpopulation (i.e. non-FSTSA) contained the association of interest and the other did not, we analysed these two subpopulations separately. Consequently, we produced stratified estimates of the effect of OHS utilisation on LTSA for those with no FSTSA and those with FSTSA separately.

Proportional hazards assumption was tested with Schoenfeld residuals test, which showed time-dependence for a violation of proportionality for FSTSA but not for the OHS utilisation variables. The result indicates that the propensity for LTSA due to mental disorders was higher within four months after FSTSAs 6th short-term SA spell. We present both direct and time-varying effects for this variable. The former represents the hazard ratio (HR) of the variable after the first four months of the follow-up, and the latter represents an increase of hazard for the first-four months of the follow-up.

Sensitivity analyses were performed to confirm the robustness of the study estimates. We replicated the results from the main analysis with estimates gained from logistic regression analyses using a dichotomised outcome. The results from these sensitivity analyses are presented in the form of marginal effects (Table 3 in Appendix 1).

All analyses were performed using Stata 16.0 software.

### Ethical considerations

The ethics committee of the Department of Public Health, the University of Helsinki and the health authorities of the City of Helsinki have approved the HHS study. The City of Helsinki and the register holders have given permission for data linkage.

## Results

### Descriptive results

**Fig. 1:**
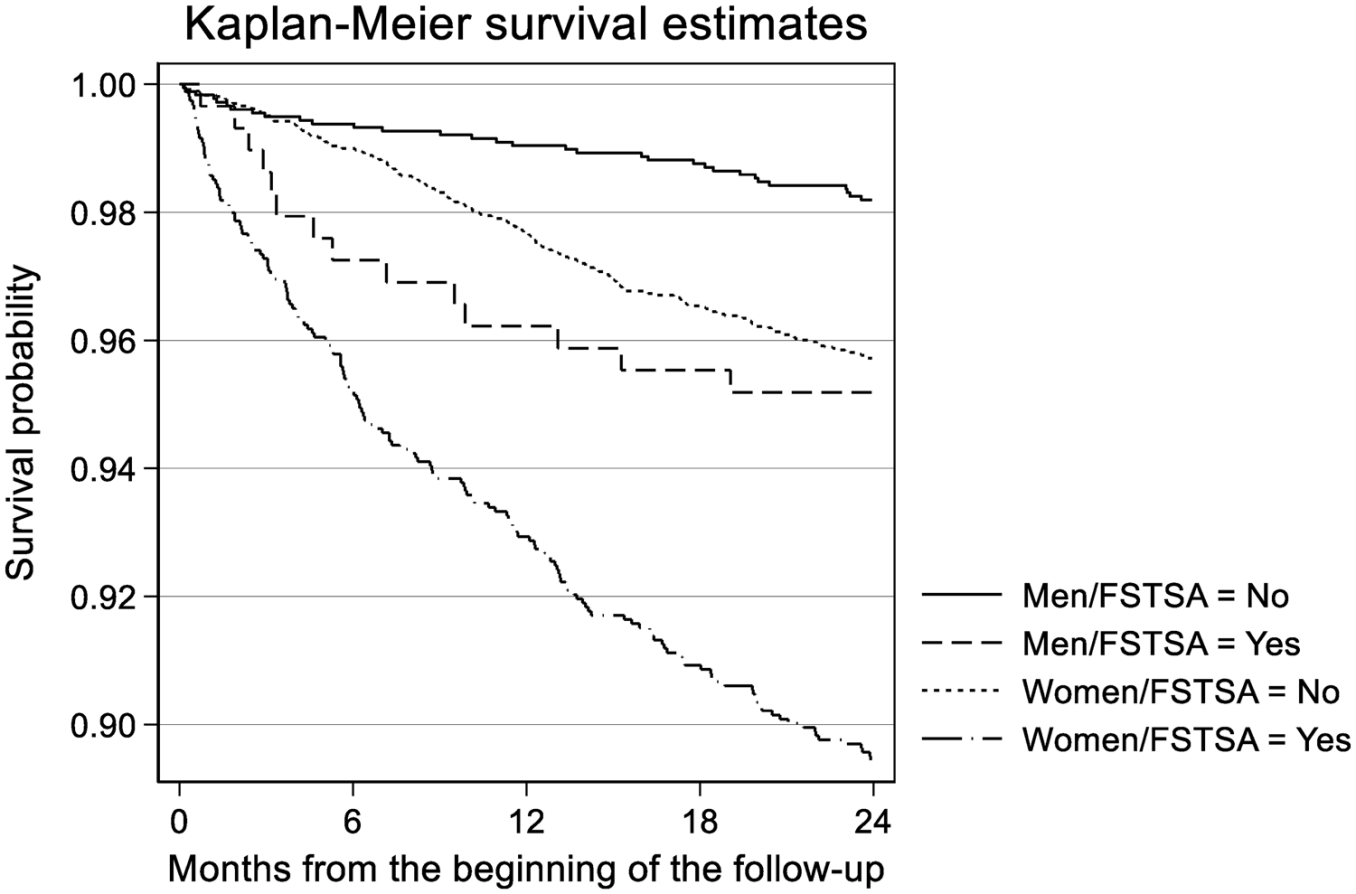
Gender differences in the association between frequent short-term sickness absence (≥ 6 spells in one year) (FSTSA) and long-term sickness absence due to mental disorders, presented as Kaplan–Meier survival curves stratified by gender and the exposure to frequent short-term sickness absence

Our sample included 8,282 observations representing 410 LTSA spells due to mental disorders. Among the 6,448 employees with no FSTSA and 1,834 with FSTSA, 3.6% of non-FSTSAs and 9.7% of FSTSAs had had a LTSA due to mental disorders during the average of 23 months follow-up (Table 1). The proportion of employees with at least one OHS primary care visit in the first-4 months of follow-up was 19% among those with non-FSTSA and 38% among those with FSTSA. Those with the highest number of OHS primary care visits both before and after the beginning of the follow-up had the highest prevalence of LTSA due to mental disorders. The relationship followed a dose–response pattern except for OHS utilisation during the follow-up among those with FSTSA. FSTSA was about twice as common in women than in men. Compared to men, among both non-FSTSA and FSTSA groups, women had only slightly higher levels of OHS utilisation but substantially higher incidence of LTSA due to mental disorders during the follow-up. The Kaplan–Meier survival curves stratified by gender and FSTSA status provide a visual representation indicating women with FSTSA to have the highest prevalence of LTSA due to mental disorders (Fig. 1).

**Table 1:** Distributions of study covariates, the proportion of participants with occupational health service (OHS) primary care visits during the one-year baseline period and first-four months of follow-up, and long-term sickness absence (LTSA) during the follow-up among the employees of the City of Helsinki, by exposure to frequent short-term sickness absence (FSTSA)

	No frequent short SAs					Frequent short SAs			
	Subjects	OHS visits, baseline		OHS visits, follow-up	SA, follow-up	Subjects	OHS visits, baseline	OHS visits, follow-up	SA, follow-up
Subjects	*n* (%)	*n* (%)		*n* (%)	*n* (%)	*n* (%)	*n* (%)	*n* (%)	*n* (%)
All	6448 (100.0)	3080 (47.8)		1195 (18.5)	232 (3.6)	1834 (100.0)	1227 (66.9)	695 (37.9)	178 (9.7)
Gender									
Men	1771 (27.5)	811 (45.8)		324 (18.3)	32 (1.8)	291 (15.9)	182 (62.5)	103 (35.4)	14 (4.8)
Women	4677 (72.5)	2269 (48.5)		871 (18.6)	200 (4.3)	1543 (84.1)	1045 (67.7)	592 (38.4)	164 (10.6)
Age									
18–24	1114 (17.3)	494 (44.3)		187 (16.8)	42 (3.8)	477 (26.0)	300 (62.9)	188 (39.4)	52 (10.9)
25–29	2571 (39.9)	1247 (48.5)		488 (19.0)	79 (3.1)	749 (40.8)	510 (68.1)	281 (37.5)	73 (9.7)
30–34	2763 (42.9)	1339 (48.5)		520 (18.8)	111 (4.0)	608 (33.2)	417 (68.6)	226 (37.2)	53 (8.7)
Occupational class									
Managers or professionals	1498 (23.2)	707 (47.2)		272 (18.2)	35 (2.3)	143 (7.8)	110 (76.9)	54 (37.8)	11 (7.7)
Semi-professionals	1698 (26.3)	824 (48.5)		324 (19.1)	61 (3.6)	431 (23.5)	296 (68.7)	176 (40.8)	36 (8.4)
Routine non-manual workers	2236 (34.7)	1044 (46.7)		415 (18.6)	105 (4.7)	1014 (55.3)	663 (65.4)	378 (37.3)	111 (10.9)
Manual workers	1016 (15.8)	505 (49.7)		184 (18.1)	31 (3.1)	246 (13.4)	158 (64.2)	87 (35.4)	20 (8.1)
Long SA at baseline, days									
0 days	6024 (93.4)	2810 (46.6)		1106 (18.4)	215 (3.6)	1568 (85.5)	1037 (66.1)	581 (37.1)	145 (9.2)
1–30 days	323 (5.0)	208 (64.4)		69 (21.4)	11 (3.4)	219 (11.9)	156 (71.2)	93 (42.5)	25 (11.4)
30 days or more	101 (1.6)	62 (61.4)		20 (19.8)	6 (5.9)	47 (2.6)	34 (72.3)	21 (44.7)	8 (17.0)
Short SAs at baseline, days									
0 days	477 (7.4)	203 (42.6)		102 (21.4)	6 (1.3)	0 (0.0)	0	0	0
1–13 days	4995 (77.5)	2224 (44.5)		868 (17.4)	172 (3.4)	362 (19.7)	198 (54.7)	100 (27.6)	18 (5.0)
14 days or more	976 (15.1)	653 (66.9)		225 (23.1)	54 (5.5)	1472 (80.3)	1029 (69.9)	595 (40.4)	160 (10.9)
Primary care visit at baseline									
0 visits	3368 (52.2)	0 (0.0)		363 (10.8)	100 (3.0)	607 (33.1)	0 (0.0)	98 (16.1)	45 (7.4)
1–2 visit	1960 (30.4)	1960 (100)		447 (22.8)	80 (4.1)	527 (28.7)	527 (100)	171 (32.4)	48 (9.1)
3 or more visits	1120 (17.4)	1120 (100)		385 (34.4)	52 (4.6)	700 (38.2)	700 (100)	426 (60.9)	85 (12.1)
Primary care visit during the follow-up									
0 visits	5253 (81.5)		2248 (42.8)	0 (0.0)	167 (3.2)	1139 (62.1)	630 (55.3)	0 (0.0)	108 (9.5)
1–2 visits	1062 (16.5)		716 (67.4)	1062 (100.0)	53 (5.0)	507 (27.6)	425 (83.8)	507 (100.0)	46 (9.1)
3 or more visits	133 (2.1)		116 (87.2)	133 (100.0)	12 (9.0)	188 (10.3)	172 (91.5)	188 (100.0)	24 (12.8)

^a^FSTSA six short sickness absence spells occurring over a one-year time-period

^b^Primary care visits at occupational health service

Differences related to occupational class were observed. The propensity for FSTSA and LTSA due to mental disorders during the follow-up were highest among routine non-manual workers. As expected, higher disease burden at baseline period also predicted both FSTSA and LTSA due to mental disorders during the follow-up. For example, 38% of those with FSTSA had three or more primary health care visits at baseline, whereas the corresponding figure for those with non-FSTSA spells was 17%. Also, the number of SA days at baseline predicted the outcome in a dose–response manner among those with FSTSA.

FSTSA predicted LTSA due to mental disorders (Table 2). The age and gender adjusted HR for those with FSTSA spells was 2.06 (95% CI 1.64, 2.60), with interaction coefficient of 2.53 (95% CI 1.58, 4.03) indicating a substantially higher association during the first four months of the follow-up (Model 1). Adjusting for OHS baseline visits lowered the HRs for FSTSA to 1.87 (95% CI 1.48, 2.37) with the time-interaction coefficient remaining unchanged (Model 2). Adjusting for occupational class slightly attenuated the estimates (Model 3), whilst additional adjustment for disease burden at baseline period decreased the estimates more considerably (Model 4). The adjustments for baseline disease burden at baseline period (measured as accumulation of past SA days and OHS utilisation at baseline) in the full model lowered the HRs for FSTSA to 1.41 (95% CI 1.36, 2.19) with the time-interaction coefficient remaining unchanged.

**Table 2:** Hazard ratios with 95% confidence intervals for long-term sickness absence (LTSA) due to mental disorders for those with frequent short sickness absence (FSTSA) spells and for those with primary care visits at occupational health service (OHS) among the employees of the City of Helsinki

Predictors	Model 1^a^	Model 2^b^	Model 3^c^	Model 4^d^
FSTSA in 1 year				
No	1.00	1.00	1.00	1.00
Yes	2.06 (1.64–2.60)	1.87 (1.48–2.37)	1.73 (1.36–2.19)	1.41 (1.36–2.19)
Interaction-term^e^	2.53 (1.58–4.03)	2.52 (1.58–4.02)	2.52 (1.58–4.02)	2.52 (1.58–4.02)
Primary care visits at OHS in 1 year				
0 visits	1.00	1.00	1.00	1.00
1–2 visits	1.40 (1.10–1.77)	1.31 (1.03–1.66)	1.33 (1.05–1.69)	1.27 (1.05–1.69)
3 or more visits	2.04 (1.61–2.58)	1.63 (1.28–2.07)	1.62 (1.27–2.06)	1.45 (1.27–2.06)
Primary care visits at OHS during the follow-up among persons with no FSTSA^f^ (< 6 short-term spells)				
0 visits	1.00	1.00	1.00	1.00
1–2 visits	1.57 (1.15–2.14)	1.47 (1.07–2.02)	1.47 (1.08–2.02)	1.47 (1.08–2.02)
3 or more visits	3.02 (1.68–5.42)	2.67 (1.47–4.86)	2.66 (1.46–4.84)	2.79 (1.46–4.84)
Primary care visits at OHS during the follow-up among persons with FSTSA^g^ (≥ 6 short-term spells)				
0 visits	1.00	1.00	1.00	1.00
1–2 visits	0.90 (0.64–1.28)	0.75 (0.52–1.09)	0.76 (0.53–1.10)	0.73 (0.53–1.10)
3 or more visits	1.34 (0.86–2.09)	1.06 (0.66–1.69)	1.06 (0.66–1.69)	1.02 (0.66–1.69)

^a^Model 1 Predictor + Age + Gender

^b^Model 2 Model 1 + FTSTA in 1 year + Primary care visits at OHS in 1 year

^c^Model 3 Model 2 + Occupational Class

^d^Model 4 Model 2 + disease burden at the baseline period

^e^Interaction term for time and variable. Coefficient represents the altered risk for the first four months of follow-up after which the risk is proportional as represented in the main coefficient

^f^Persons with no frequent short-term sickness absence (< 6 spells in one year)

^g^Persons with frequent short-term sickness absence (≥ 6 spells in one year)

*OHS* occupational health service

Primary care visits at OHS at baseline produced broadly similar results to those with FSTSA. The age and gender adjusted HR was 1.40 (95% CI 1.11, 1.77) for those with 1–2 OHS visits, and 2.04 (95% CI 1.61, 2.58) for those with three or more visits. In the model adjusting for FSTSA, the HRs for the OHS use at baseline were 1.31 (95% CI 1.03, 1.66) and 1.63 (95% CI 1.28, 2.07), correspondingly. Models 3 and 4 had only marginal effect on the estimates.

Primary care visits at OHS during the follow-up were associated with LTSA due to mental disorders only in those with no FSTSA. In this group the estimates for those with three or more OHS visits ranged from 3.02 (95% CI 1.68, 5.42) in model 1 to 2.67 (95% CI 1.47, 4.86) in model 2, with only small changes in models 3 and 4. The corresponding figures for the FSTSA group were 1.34 (95% CI 0.86, 2.09) and 1.06 (95% CI 0.66, 1.69).

Sensitivity analyses using the dichotomised outcome and logistic regression analyses produced estimates comparable with the presented results thus indicating robustness of our findings (Table 3 in Appendix 1). The marginal effect estimates indicate that obtainable reduction of LTSA rates to be 2.69–5.27 percentage points for FSTSA, 1.59–3.51 percentage points for three or more baseline OHS visits, and 5.05–5.65 percentage points for three or more OHS visits after FSTSA.

## Discussion

In this study, we found that frequent short-term sickness absence (FSTSA) due to all causes was associated with subsequent long-term sickness absence (LTSA) (over 11 calendar days) due to mental disorders. In addition, we found that increased primary care use in occupational health services (OHS), measured as the number of visits at one-year baseline period, was associated with LTSA due to mental disorders independently of FSTSA. These associations remained statistically significant, after controlling for the studied covariates, i.e. occupational class and disease burden at baseline period. The results point to the following interpretations. First, both FSTSA and increased OHS utilisation may be interpreted as indicators of unobserved processes of health degradation leading to LTSA due to mental disorders, and that the use of primary care utilisation data may be used to identify a larger proportion of individuals at risk of LTSA due to mental disorders compared with monitoring FSTSA data solely. Second, we found that occupational class had modest-to-none influence to the estimates, i.e. despite differences in the overall distribution of FSTSA and LTSA due to mental disorders, as seen in the descriptive results, there were no substantive differences in how FSTSA or OHS utilisation associated with LTSA due to mental disorders across occupational classes. While the processes leading to LTSA due to mental disorder may be dependent on different sociodemographic and occupational factors, such as gender or the differences of job-related characteristics of different occupations, FSTSA and OHS use provide indication of these processes consistently across these population groups. Third, the changes in estimates after controlling for the disease burden at baseline period suggest that FSTSA and OHS identify both individuals with and without accumulated sickness absence days. Fourth, in the stratified analysis of OHS use after short-term SA among those with and without FSTSA, we did not find association between the OHS use and LTSA due to mental disorders among those with FSTSA, whereas the association was found among those with no FSTSA. The result may be explained by the partial correlation between FSTSA and OHS use but, also, we find it likely that the use of OHS for obtaining a medical certificate for SA lasting more than 3 days is more prevalent among those with FSTSA. We acknowledge that as OHS use during the follow-up is measured during the same period as the outcome, further studies are required to confirm these associations. Finally, the marginal effects estimates that were presented in sensitivity analyses indicate that the potential for reduction in LTSA rates among FSTSA and frequent OHS user populations is discernible.

Short-term SA spells are relatively common among young employees. Only few had none of them at all, and slightly less than one quarter of young employees had six or more short-term SA spells in a year. This led us to use six spells threshold for FSTSA, which is higher than the usually used threshold (Notenbomer et al. 2019; Koopmans et al 2008; Sumanen et al. 2017). In line with previous studies, we found that accumulation of short-term SA predicted subsequent LTSA due to mental disorders (Notenbomer et al 2019; Koopmans et al 2008; Laaksonen et al. 2013; Sumanen et al. 2017). Our results complement these results by finding that employees with FSTSA were also more likely to have a higher level of use of OHS both before and after a short-term SA spell, which in turn was associated with an increased risk for LTSA due to mental disorders. Our results provide grounds for further investigation of accumulation of short-term SA together with primary care utilisation data.

Our results point out the gendered distribution of both LTSA due to mental disorders and the preceding risk factors. We found that FSTSA spells were more common among women than among men, as was subsequent long-term SA due to mental disorders. Moreover, the highest prevalence of SA due to mental disorders was found in the female dominated occupational group of lower non-manual workers, i.e. among occupations such as child and elderly care workers. The result is in line with earlier findings on the gendered occupational statuses and their relation to the prevalence of mental disorders (Halonen et al. 2018; Lidwall et al. 2018). Our results complement these findings by showing that FSTSA and OHS may be used as risk indicators consistently across these occupational classes. However, due to the limitations of the register-based data we could not differentiate between gender-related biological, individual or work-related factors, such as experienced mental distress, poor support from superiors (Foss et al. 2010), adverse psychosocial work environments (Lidwall et al. 2018) or emotional demands (Framke et al. 2019) that are risk factors more prevalent in female dominated health and social care occupations. Despite this limitation, a balanced approach between health care-based and workplace-based interventions in prevention of LTSA due to mental disorders is likely to be required.

We found that the employee’s disease burden at baseline period, measured as accumulation of past SA days and OHS utilisation at baseline, turned out to be a significant factor explaining the link between repeated short-term SA and LTSA. Individuals who, in addition to FSTSA had longer spells of previous SA and high levels of OHS use at baseline were more likely to experience LTSA due to mental disorders. Individuals who, in addition to FSTSA spells, also had preceding longer spells of LTSA due to other reasons than mental disorders or more accumulated days during short-term SA or high levels of OHS use were in the highest risk of experiencing subsequent LTSA due to mental disorders. Therefore, our analysis suggests that FSTSA spells, alongside with other indicators that are retrievable from electronic records, may accurately indicate altered levels of mental burden and processes leading to LTSA due to mental disorders. Our results thus highlight the importance of sensitivity to accumulation of short-term SA together with other indicators of cumulative disease burden at baseline period. As the symptoms at early stage could be more easily treated, further investigation is needed to explore whether the measures applied in this study could be used as proxies for the symptoms of common mental disorders (Framke et al. 2019; Knudsen et al. 2013) or mental well-being and work capacity (Bertilsson et al. 2015) that have also been proven to operate as risk-factors to the onset, duration and recurrence of subsequent SA.

As far as we are aware, this is the first study to explore the role of OHS primary care use in the progress from FSTSA to LTSA due to mental disorders. The registers of the largest employer of Finland were used in this study. The registers are comprehensive and reliable, cover all employees, information of OHS visits and all SA spells. Thus, this study avoids a common limitation of OHS utilisation studies that are based on cross-sectional data, or limited samples with data on only those who have used the services. However, limitations of the data include the lack of information of diagnoses regarding short-term SA. Despite this limitation we found that the routine OHS utilisation and SA data could be used to identify those who will have subsequent LTSA due to mental disorders. The study also indicates that OHS visits alone are not sufficient in preventing LTSA and resources might be used in potentially supportive actions, such as cognitive behavioural therapy and problem-solving therapy (Lexis et al. 2011).

Previous evidence underlines the importance of monitoring the accumulating short-term SA in order to utilise preventive measures at early stages, already before more severe work disability leading to longer SA occurs (Kivimäki et al. 2004; Koopmans et al. 2008). The Finnish OHS and workplaces have widely reacted to this, and the FSTSA monitoring is generally a part of their early support models. Early support models are one of the key elements of statutory work ability management performed in co-operation between OHS and workplaces. Monitoring FSTSA is possibly a very feasible preventive measure and also an equal way of implementing early support when and where needed, as the operating models are written policies that are applied to all employees.

A cautionary remark on the generalisability of the results is warranted. For example, we studied a population consisting of municipal employees in a particular OHS context. However, the OHS system is the main primary care service provider for the working population in Finland (Ikonen et al. 2013; Virtanen and Mattila 2011), and the principle of primary care use is similar as in the general practice in most other Western countries. Our findings obtained from a reliable longitudinal register data encourage integrating indicators from primary health care services in sickness absence studies to inform the programs aiming at preventing and mitigating the risks for LTSA due to mental disorders.

## Conclusions

Both frequent short-term sickness absences and occupational health service utilisation were associated with subsequent long-term sickness absence due to mental disorders. Routinely collected data on health service utilisation could be used as a source of information for identification of individuals at risk of long-term sickness absence due to mental disorders and targeting preventive measures for them.

## Data Availability

Data are not shared due to the legal and regulatory restrictions.

## Appendix 1

See Table 3.

**Table 3:** Marginal effects (Adjusted Predictions at the Means) with 95% confidence intervals for long-term sickness absence (LTSA) due to mental disorders for those with frequent short-term sickness absence (FSTSA) spells and for those with primary care visits among the employees of the City of Helsinki

Predictors	Model 1^a^	Model 2^b^	Model 3^c^	Model 4^d^
FSTSA in 1 year				
No	3.45 (3.00–3.90)	3.47 (3.01–3.92)	3.45 (3.00–3.91)	3.58 (3.10–4.06
Yes	8.72 (7.41–10.03)	7.97 (6.69–9.25)	7.36 (6.12–8.60)	6.27 (4.97–7.57)
Primary care visits at OHS in 1 year				
0 visits	3.44 (2.87–4.00)	3.47 (2.90–4.04)	3.38 (2.82–3.95)	3.48 (2.90–4.06)
1–2 visits	4.76 (3.92–5.59)	4.52 (3.71–5.32)	4.47 (3.66–5.27)	4.40 (3.60–5.20)
3 or more visits	6.95 (5.78–8.11)	5.64 (4.60–6.68)	5.48 (4.46–6.50)	5.07 (4.08–6.06)
Primary care visits at OHS during the follow-up among persons with no FSTSA^e^ (< 6 short-term spells)				
0 visits	2.96 (2.49–3.43)	2.97 (2.50–3.45)	2.87 (2.40–3.34)	2.89 (2.42–3.36)
1–2 visits	4.63 (3.39–5.88)	4.37 (3.15–5.58)	4.22 (3.04–5.41)	4.23 (3.04–5.42)
3 or more visits	8.61 (3.90–13.3)	7.74 (3.38–12.11)	7.54 (3.26–11.82)	7.94 (3.44–12.44)
Primary care visits at OHS during the follow-up among persons with FSTSA^f^ (≥ 6 short-term spells)				
0 visits	9.21 (7.50–10.90)	9.67 (7.88–11.46)	9.60 (7.81–11.38)	9.43 (7.64–11.21)
1–2 visits	8.60 (6.18–11.03)	7.62 (5.33–9.92)	7.64 (5.34–9.94)	7.32 (5.08–9.56)
3 or more visits	12.55 (7.80–17.30)	10.61 (6.29–14.92)	10.54 (6.24–14.84)	10.07 (5.90–14.23)

The estimates were derived from logistic regression analysis

^a^Model 1 Predictor + Age + Gender

^b^Model 2 Model 1 + FTSTA in 1 year + Primary care visits at OHS in 1 year

^c^Model 3  Model 2 + Occupational Class

^d^Model 4  Model 2 + disease burden at the baseline period

^e^Persons with no frequent short-term sickness absence (< 6 spells in one year)

^f^Persons with frequent short-term sickness absence (≥ 6 spells in one year)

## References

[CR1] Airaksinen J, Jokela M, Virtanen M, Oksanen T, Koskenvuo M, Pentti J, Vahtera J, Kivimäki M (2018). Prediction of long-term absence due to sickness in employees: development and validation of a multifactorial risk score in two cohort studies. Scand J Work Environ Health.

[CR2] Baumann AE (2007). Stigmatization, social distance and exclusion because of mental illness: the individual with mental illness as a stranger. Int Rev Psychiatry.

[CR3] Bertilsson M, Vaez M, Waern M, Ahlborg G, Hensing G (2015). A prospective study on self-assessed mental well-being and work capacity as determinants of all-cause sickness absence. J Occup Rehabil.

[CR4] Bultmann U, Huibers MJ, van Amelsvoort LP, Kant I, Kasl SV, Swaen GM (2005). Psychological distress, fatigue and long-term sickness absence: prospective results from the Maastricht Cohort Study. J Occup Environ Med.

[CR5] Foss L, Gravseth HM, Kristensen P, Claussen B, Mehlum IS, Skyberg K (2010). Risk factors for long-term absence due to psychiatric sickness: a register-based 5-year follow-up from the Oslo health study. J Occup Environ Med.

[CR6] Framke E, Sørensen JK, Nordentoft M, Johnsen NF, Garde AH, Pedersen J, Madsen IEH, Rugulies R (2019). Perceived and content-related emotional demands at work and risk of long-term sickness absence in the danish workforce: a cohort study of 26 410 Danish employees. Occup Environ Med.

[CR7] Gili M, Luciano JV, Serrano MJ, Jiménez R, Bauza N, Roca M (2011). Mental disorders among frequent attenders in primary care. J Nerv Ment Dis.

[CR8] Gissler M, Haukka J (2004). Finnish health and social welfare registers in epidemiological research. Norsk Epidemiologi.

[CR9] Halonen JI, Koskinen A, Varje P, Kouvonen A, Hakanen JJ, Väänänen A (2018). Mental health by gender-specific occupational groups: profiles, risks and dominance of predictors. J Affect Disord.

[CR10] Harkko J, Sumanen H, Pietiläinen O, Piha K, Mänty M, Lallukka T, Rahkonen O, Kouvonen A (2020). Socioeconomic differences in occupational health service utilization and sickness absence due to mental disorders: a register-based retrospective cohort study. Int J Environ Res Public Health.

[CR12] Ikonen A, Räsänen K, Manninen P, Rautio M, Husman P, Ojajärvi A, Alha P, Husman K (2013). Use of health services by Finnish employees in regard to health-related factors: the population-based health 2000 study. Int Arch Occup Environ Health.

[CR13] Kela Statistical Yearbook of the Social Insurance Institution 2017 (2020) Available online: https://helda.helsinki.fi/bitstream/handle/10138/270222/Kelan_tilastollinen_vuosikirja_2017.pdf?sequence=21&isAllowed=y. Accessed 31 Jan 2020

[CR14] Kivimäki M, Forma P, Wikström J, Halmeenmäki T, Pentti J, Elovainio M, Vahtera J (2004). Sickness absence as a risk marker of future disabilitypension: the 10-town study. J Epidemiol Community Health.

[CR15] Knapstad M, Øverland S, Henderson M, Holmgren K, Hensing G (2014). Shame among long-term sickness absentees: correlates and impact on subsequent sickness absence. Scand J Public Health.

[CR16] Knudsen AK, Harvey SB, Mykletun A, Øverland S (2013). Common mental disorders and long-term sickness absence in a general working population The Hordaland Health Study. Acta Psychiatr Scand.

[CR17] Koopmans PC, Roelen CA, Groothoff JW (2008). Risk of future sickness absence in frequent and long-term absentees. Occup Med.

[CR18] Lexis MA, Jansen NW, Huibers MJ, van Amelsvoort LG, Berkouwer A, Tjin A, Ton G, van den Brandt PA, Kant I (2011). Prevention of long-term sickness absence and major depression in high-risk employees: a randomised controlled trial. Occup Environ Med.

[CR19] Laaksonen M, He L, Pitkäniemi J (2013). The durations of past sickness absence spells predict future absence episodes. J Occup Environ Med.

[CR20] Lahelma E, Aittomäki A, Laaksonen M (2013). Cohort profile: the helsinki health study. Int J Epidemiol.

[CR21] Lidwall U, Bill S, Palmer E, Olsson Bohlin C (2018). Mental disorder sick leave in Sweden: a population study. Work.

[CR22] Mauramo E, Lallukka T, Lahelma E, Pietiläinen O, Rahkonen O (2018). Common mental disorders and sickness absence: a register-linkage follow-up study among finnish municipal employees. J Occup Environ Med.

[CR23] Nicholson PJ (2018). Common mental disorders and work. Br Med Bull.

[CR25] Notenbomer A, van Rhenen W, Groothoff JW, Roelen CAM (2019). Predicting long-term sickness absence among employees with frequent sickness absence. Int Arch Occup Environ Health.

[CR26] Reho T, Atkins S, Talola N, Viljamaa M, Sumanen M, Uitti J (2018). Occasional and persistent frequent attenders and sickness absences in occupational health primary care: a longitudinal study in Finland. BMJ Open.

[CR27] Reho T, Atkins S, Talola N, Viljamaa M, Sumanen M, Uitti J (2020). Frequent attenders at risk of disability pension: a longitudinal study combining routine and register data. Scand J Public Health.

[CR29] Saarni SI, Suvisaari J, Sintonen H, Pirkola S, Koskinen S, Aromaa A, Lönnqvist J (2007). Impact of psychiatric disorders on health-related quality of life: general population survey. Br J Psychiatry.

[CR30] Schouten LS, Bültmann U, Heymans MW, Joling CI, Twisk JW, Roelen CA (2016). Shortened version of the work ability index to identify workers at risk of long-term sickness absence. Eur J Public Health.

[CR31] Smits FT, Brouwer HJ, Zwinderman AH, Mohrs J, Schene AH, Van Weert HC (2014). Why do they keep coming back? Psychosocial etiology of persistence of frequent attendance in primary care: A prospective cohort study. J Psychosom Res.

[CR32] Stansfeld SA, Fuhrer R, Head J (2011). Impact of common mental disorders on sickness absence in an occupational cohort study. Occup Environ Med.

[CR33] Sumanen H, Pietiläinen O, Lahelma E, Rahkonen O (2017). Short sickness absence and subsequent sickness absence due to mental disorders—a follow-up study among municipal employees. BMC Public Health.

[CR11] van Hoffen MFA, Norder G, Twisk JWR, Roelen CAM (2019). Development of prediction models for sickness absence due to mental disorders in the general working population. J Occup Rehabil.

[CR34] Virtanen P, Mattila K (2011). Työterveyshuollon potilas käy myös terveyskeskuksessa, tosin harvoin. [Patients of occupational health care also visit health centres, albeit infrequently] (English summary). Suom Laakaril.

